# Pancancer Analyses Reveal Genomics and Clinical Characteristics of the SETDB1 in Human Tumors

**DOI:** 10.1155/2022/6115878

**Published:** 2022-05-23

**Authors:** Xin Lin, Min Xiao, Zhitao Chen, Chenchen Ding, Ting Zhang, Qiyong Li

**Affiliations:** ^1^Department of Hepatobiliary Surgery, Shulan (Hangzhou) Hospital Affiliated to Zhejiang Shuren University Shulan International Medical College, Hangzhou, China; ^2^School of Medicine, Zhejiang University, Hangzhou, China

## Abstract

**Background:**

Malignant tumor is one of the most common diseases that seriously affect human health. The prior literature has reported the biological function and potential therapeutic targets of SET domain bifurcated histone lysine methyltransferase 1 (SETDB1) as an oncogene. However, SETDB1 has rarely been analyzed from a pan-cancer perspective.

**Methods:**

Bioinformatics analysis tools and databases, including GeneCards, National Center for Biotechnology Information (NCBI), UniProt, Illustrator for Biological Sequences (IBS), Human Protein Atlas (HPA), GEPIA, TIMER2, Sangerbox 3.0, UALCAN, Kaplan-Meier (K-M) plotter, cBioPortal, Catalogue Of Somatic Mutations In Cancer (COSMIC), PhosphoSitePlus, TISIDB, STRING, and GeneMANIA, were utilized to clarify the biological functions and clinical significance of SETDB1 from a pan-cancer perspective.

**Results:**

In this study, the pan-cancer analysis demonstrated that SETDB1 showed significantly differential expression in most tumor tissues and paracancerous tissues, and SETDB1 expression was associated with clinicopathological features and clinical prognosis. We also found that SETDB1 mutations occurred in most tumors and were related to tumorigenesis. In addition, DNA methylation of SETDB1 primarily occurred at the cg10444928 site and was associated with prognosis in several human tumors. The predicted phosphorylation site of SETDB1 was Ser1006. We found that SETDB1 was significantly related to the specific tumor-infiltrating immune cell populations and expression of clinically targetable immune checkpoints and may be a promising immunotherapy target. The Gene Ontology (GO) and Kyoto Encyclopedia of Genes and Genomes (KEGG) analyses also indicated that SETDB1 may function as crucial regulator in carcinogenesis of human cancers.

**Conclusions:**

SETDB1 is an important oncogene involved in tumorigenesis and tumor progression through different biological mechanisms. Furthermore, SETDB1 may be a potential therapeutic target for cancer treatment.

## 1. Introduction

Malignant tumor is becoming a major disease endangering human health [1]. At present, there are no curative strategies for malignant tumors. Antitumor therapies, including radical surgical resection, radiofrequency ablation, transplantation, chemotherapy, immunotherapy, and targeted therapy, have been developed [2]. However, the overall survival (OS) for patients with cancer, especially pancreatic adenocarcinoma (PAAD), lung adenocarcinoma (LUAD), and breast invasive carcinoma (BRCA), remains low because of the complexity and heterogeneity of tumorigenesis [3–5]. Germline mutations caused by abnormal activation and expression of oncogenes have also been confirmed as major inducements of tumorigenesis [6]. The investigation of epigenetic changes, expression levels, potential molecular basis, and clinical significance of oncogene can help understand the mechanisms of tumorigenesis and improve the treatment of various cancers.

SET domain bifurcated histone lysine methyltransferase 1 (SETDB1) protein, also known as ERG-associated protein with SET domain (ESET), KG1T, KMT1E, TDRD21, and H3-K9-HMTase4, is a member of the SET family involved in chromatin gene silencing, chromatin remodeling, transcriptional suppression, and histone methylation in cells [7, 8]. The SET family also shows epigenetic regulation, participating in gene expression and function changes without altering the DNA sequence. SET family is a significant regulator of tumorigenesis and is important for tumor-targeted therapy [9, 10]. SETDB1 was first reported in 1999, and increasing studies have found that SETDB1 is significantly related to human tumorigenesis and immune cell functions [11–13]. SETDB1 is also a well-known histone H3 lysine 9 (H3K9) methyltransferase that associates with methylation in various euchromatic regions, which causes gene silencing [12]. Therefore, it is important to conduct a comprehensive genomic analysis of SETDB1 and explore its relation with clinical outcome and potential target of oncotherapy in human malignant tumors.

In this study, for the first time, we conducted a structure/function pan-cancer analysis of SETDB1 based on several online databases to explore its oncogenic role and clinical significance in various cancers.

## 2. Material and Methods

### 2.1. Omics Analysis of SETDB1

Firstly, we acquired the chromosome localization, coding sequence (CDS), and exon counts of SETDB1 based on the GeneCards database (https://www.genecards.org/). Subsequently, the biological information of the SETDB1 gene and its encoded six protein isoforms was obtained from the “gene” and “protein” module of the National Center for Biotechnology Information (NCBI) (https://www.ncbi.nlm.nih.gov/), with its 3D (three-dimensional) protein structure explored in the UniProt database (https://www.uniprot.org/). In addition, the CDS in nucleotide sequence and conserved domains in amino acid sequence were visualized using Illustrator for Biological Sequences (IBS, version 1.0) [14] (http://ibs.biocuckoo.org/). The position of conserved domains of histone-lysine N-methyltransferase SETDB1 isoform 1 protein was obtained from the “HomoloGene” of NCBI. Conserved amino acid sequences encoded by SETDB1 and phylogenetic tree of SETDB1 family were explored by Constraint-based Multiple Alignment Tool (https://www.ncbi.nlm.nih.gov/tools/cobalt/) in the NCBI. Finally, the distribution of SETDB1 protein was obtained from Human Protein Atlas (HPA) (https://www.proteinatlas.org/) database.

### 2.2. Gene Expression Analysis

The expression levels of mRNA and encoded protein of SETDB1 in normal tissues were obtained from several online databases, including GEPIA [15] (http://gepia.cancer-pku.cn/), HPA, and the University of California, Santa Cruz (UCSC) Xena browser (https://xenabrowser.net). The datasets were derived from The Cancer Genome Atlas (TCGA) (https://www.cancer.gov/) and Genotype-Tissue Expression Project (GTEx) (https://www.genome.gov/). Additionally, the immunohistochemical (IHC) staining and hematoxylin-eosin (H&E) staining displayed the top five significantly expressed tissues of SETDB1. In this study, we also compared the mRNA expression of the SETDB1 gene in different cell lines and single cell specificity. TIMER2 (http://timer.cistrome.org/), Sangerbox 3.0 (http://vip.sangerbox.com/), UALCAN (http://ualcan.path.uab.edu/), and GEPIA2 [16] (http://gepia2.cancer-pku.cn) databases were also applied to explore and verify the SETDB1 differential expression levels in tumor tissues and paracancerous tissues.

### 2.3. Clinicopathological Features Analysis

The “Pathological Stage Plot” module of GEPIA2 was applied to assess the relationship between the SETDB1 gene expression and cancer stage based on TCGA. *P* < 0.05 was set as the significance threshold. We also used the UALCAN database to explore the relationship between the SETDB1 mRNA expression level and clinicopathological stage, including the within-stage correlation. Sangerbox 3.0 was applied to confirm the connection between the SETDB1 mRNA transcription level and other clinicopathological features, including TNM classification and clinicopathological grade.

### 2.4. Survival Analysis

GEPIA2 is also an available comprehensive prognosis analysis database, and its target gene was used as input for survival analysis in various human cancers. In this study, we first used the “Survival Map” module of GEPIA2 to explore the OS and disease-free survival (DFS) significance map data of SETDB1 among all tumors from TCGA datasets. The patients with cancer were divided into the high- and low-expression subgroups according to the median expression levels of SETDB1. Subsequently, the “survival analysis” module of GEPIA2 was used to draw the significance of the Kaplan-Meier (K-M) curves for patients with cancer, and *P* < 0.05 was set as the significance threshold. Furthermore, the Cox analyses based on Sangerbox 3.0 database were performed for disease-specific survival (DSS) and progression-free interval (PFI) of SETDB1 across various cancers samples, and the results were displayed by a forest plot. Then, we explored the prognostic value of SETDB1 in ovarian cancer, liver hepatocellular carcinoma (LIHC), LUAD, and BRCA based on the K-M plotter [17] (http://kmplot.com/analysis/). The survival datasets were derived from TCGA and Gene Expression Omnibus (GEO) (https://www.ncbi.nlm.nih.gov/geo/).

### 2.5. Genetic Alteration Analysis

A total of 32 studies containing 10967 samples were selected from the “TCGA PanCancer Atlas Studies” module of cBioPortal (https://www.cbioportal.org) online database. The genetic alteration levels of SETDB1 were further explored using 110443 samples with mutation data. The totality genetic alteration samples of SETDB1 across TCGA tumors were generated from the “OncoPrint” module of cBioPortal. According to the “Cancer Types Summary” module of cBioPortal, the alteration frequency, number of genetic mutations, type of SETDB1 mutations, and copy number variation (CNV) in each tumor type were analyzed. The mutated site information of SETDB1 was shown in the amino acid sequence containing conserved domain sites and the 3D structure by the “Mutations” module. The Sangerbox 3.0 online serve was used to comprehensively analyze the mutational landscape of SETDB1. The GSCA database (http://bioinfo.life.hust.edu.cn/GSCA/#/) was used to analyze the CNV percentage in each cancer and the relationship between SETDB1 expression and CNV. The Catalogue Of Somatic Mutations In Cancer (COSMIC) (https://cancer.sanger.ac.uk/cosmic) is also a comprehensive alteration analysis database for exploring the mutation of SETDB1 in human cancers.

### 2.6. Methylation and Protein Phosphorylation Analysis

We first assessed the differential expression of SETDB1 promoter methylation in tumor tissues and normal tissues with the UALCAN online database. Furthermore, the relationship between SETDB1 expression and RNA modification-related genes was explored using the Sangerbox 3.0 online service. The MethSurv [18] (https://biit.cs.ut.ee/methsurv/) online database was used to obtain the relative expression level of single CpGs of SETDB1 methylation and their prognostic value. MethSurv database is specifically designed to compare the relative expression level of a single CpG and perform multivariable survival analysis using DNA methylation data. The prognostic value of single CpG of SETDB1 in 25 cancers was also assessed using the “all cancers” and “single CpG” modules of the MethSurv database.

Subsequently, the PhosphoSitePlus (version 6.6.0.2, https://www.phosphosite.org/) was used to investigate the protein phosphorylation sites of SETDB1 in amino acid sequence. The UALCAN online database was also used to compare SETDB1 phosphorylation levels between tumor tissues and paracancerous tissues. The protein phosphorylation data were sourced from the Clinical Proteomic Tumor Analysis Consortium (CPTAC) (https://proteomics.cancer.gov/programs/cptac), and BRCA, glioblastoma multiforme (GBM), PAAD, head and neck squamous cell carcinoma (HNSC), and LUAD.

### 2.7. Immune and Molecular Subtype Analysis, Immune Infiltration Analysis, and Immune Checkpoint Inhibitor-Related Gene Analysis of SETDB1

We then logged into the TISIDB [19] (http://cis.hku.hk/TISIDB/index.php) database with submitting “SETDB1” to assess the association of SETDB1 expression status with immune cell and molecular subtypes in various human cancers. The “Immune-Gene” module of the TIMER2, which is specifically designed to analyze the immune infiltration across all TCGA cancers, was applied to explore the association between the SETDB1 expression and tumor-related immune cell infiltration levels. The SETDB1 expression was related to the abundance of tumor-infiltrating cells, including cancer-associated fibroblasts (CAFs), CD8^+^ T cells, CD4^+^ T cells, regulatory T cells (Tregs), and B cells. The scatterplots were used to present the correlation between SETDB1 mRNA expression and the abundance of infiltrating CAFs. The TISIDB database was also used to analyze the association of SETDB1 expression with immune checkpoint inhibitor-related genes, including immunoinhibitor and immunostimulator. Part of the results with significant correlation was presented as scatterplots. To identify potential groups that may benefit from immunotherapy, we used the radar chart to display the association between microsatellite instability (MSI) and tumor mutational burden (TMB) in various cancers based on the Sangerbox 3.0.

### 2.8. Function and Pathway Analysis

The STRING (https://string-db.org/) database was used to explore the targeting gene-binding proteins by searching protein name “SETDB1” and organism type “Homo sapiens.” By setting parameters of STRING, the experimentally determined SETDB1-binding proteins were obtained. Using GeneMANIA (http://genemania.org/) online database, we predicted the possible function of SETDB1-related genes according to their association with genes with assigned biological functions. To clarify the functions of the target genes, the Gene Ontology (GO) functional enrichment analysis and Kyoto Encyclopedia of Genes and Genomes (KEGG) pathway analysis of SETDB1-related genes were performed by R (version R 3.6.3, https://www.r-project.org/). Furthermore, the HALLMARK terms were analyzed by Sangerbox 3.0. In order to construct the mRNA-miRNA-lncRNA network, we first predicted the miRNA targeting SETDB1 based on TargetScanHuman (Release 7.2 March 2018, http://www.targetscan.org/), mirDIP (http://ophid.utoronto.ca/mirDIP/), and miRWalk (http://mirwalk.umm.uni-heidelberg.de/). Then, the complementary sequences of SETDB1 and miRNA targeting SETDB1 were displayed using the TargetScanHuman database. Finally, the lncRNA targeting miRNAs were predicted by the LncBase Predicted v2 module of DIANA tools (http://carolina.imis.athena-innovation.gr/diana_tools/web/index.php?r=site/index).

### 2.9. Drug Sensitivity and Resistance Analysis

The drug sensitivity analysis and resistance analysis were performed based on the Genomics of Drug Sensitivity in Cancer (GDSC) (https://www.cancerrxgene.org/) database, and the volcano plot was displayed. The IC50 values of Ara-G and Bleomycin (50 *μ*M) for SETDB1 mutation were analyzed. GSCALite (http://bioinfo.life.hust.edu.cn/web/GSCALite/) is a comprehensive web-based analysis platform for gene set cancer analysis and drug sensitivity analysis. DrugBank (https://www.drugbank.com/) database was used to explore the chemical formula and structural formula for Ara-G and Bleomycin (50 *μ*M).

### 2.10. Validating Expression of SETDB1 by IHC

Six pairs of paraffin-embedded digestive system tumors, including LIHC, CHOL, COAD, ESCA, PAAD, and STAD, and corresponding adjacent tissues were collected in the Shulan (Hangzhou) Hospital. Collected tissues were embedded in paraffin and sliced into 4 *μ*m sections, then baked in an oven at 65°C for 2 hours, and hydrated. These tissues were incubated with 1 : 25 dilution of anti-SETDB1 monoclonal antibody (catalog number: KHC0067). After incubation with the anti-rabbit secondary antibody (ORIGENE) at room temperature for 1 h, diaminobenzidine (DAB) was used to reveal the color of antibody staining. Finally, the stained sections were observed under the microscope.

## 3. Results

### 3.1. Multiomics Analysis of SETDB1

**Figure 1:**
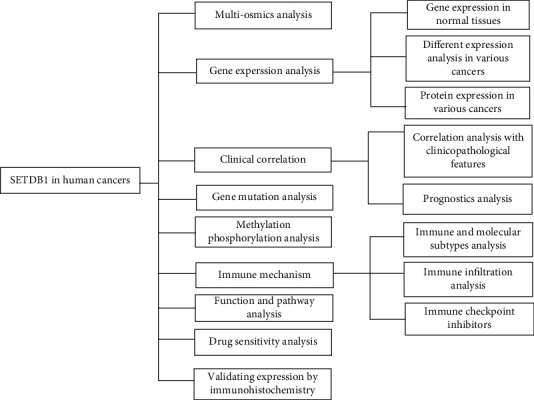
The workflow of the study.

**Figure 2:**
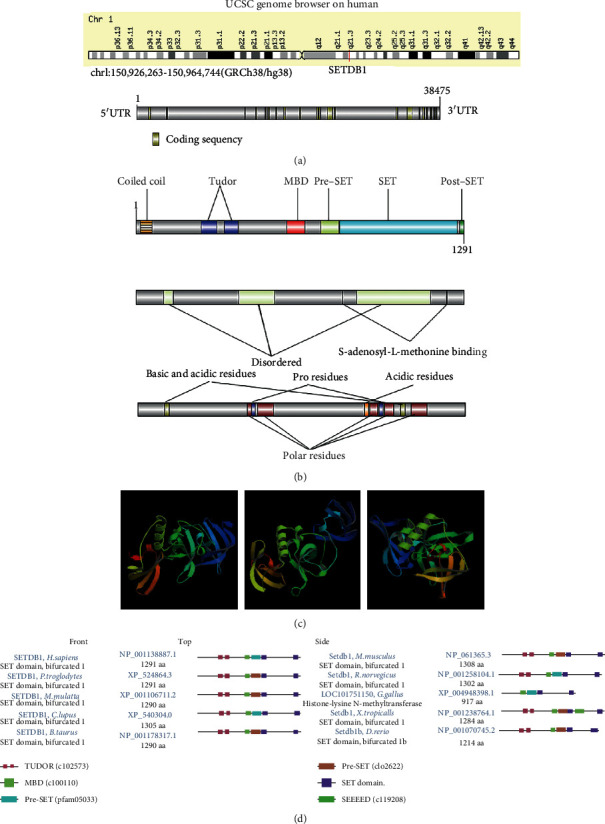
Gene structure, protein structure, and conserved domain of SETDB1. (a) Chromosome localization and gene coding sequence (CDS) of SETDB1 in human. (b) The six domains, five regions, one coiled coil, and ten nucleotide compositional biases of protein structure of SETDB1. (c) The protein structure of SETDB1 gene. (d) Conservation of SETDB1 protein among different species.

The details of the pan-cancer analysis are summarized and presented in Figure 1. This study was aimed at investigating the oncogenic role of SETDB1 in human cancers. SETDB1 (Gene ID: 9869) is located at 1q21.3 and contains 23 exons (Figure 2(a)). The CDS of SETDB1 in nucleotide sequence was displayed (Figure 2(a)). The SETDB1 encoded six protein isoforms, including histone-lysine N-methyltransferase SETDB1 isoforms 1-6, which were mainly distributed in the nucleoplasm (Figure S1A). The mRNA, protein reference sequences (Refseq), and the conserved domains of SETDB1 were summarized (Table 1). Histone-lysine N-methyltransferase SETDB1 isoform 1 was the dominant isoform and the main undertaker of histone-lysine N-methyltransferase SETDB1 functions. To better understand the biological function and structural information of histone-lysine N-methyltransferase SETDB1 protein isoform 1, structure-function analysis was conducted, and the protein domains, region, and nucleotide compositional bias were displayed. As shown in Figure 2(b), the protein structure of SETDB1 consists of six domains, five regions, one coiled coil, and ten nucleotide compositional biases. For multiple species, the SETDB1 contains six domains, including two Tudors (cl02573), MBD (cl00110), pre-SET (cl02622), SET (cl02566), post-SET, and SEEEED (cl19208) (Figure 2(d)). The pre-SET, SET, and post-SET domains are required for methyltransferase activity. Additionally, the protein 3D structure was also displayed (Figure 2(c)). According to the NCBI online database, the SETDB1 protein was conserved in different species, such as chimpanzee, rhesus monkey, dog, cow, mouse, rat, chicken, zebrafish, and frog (Figure S1B). The phylogenetic tree of SETDB1 protein was produced using the fast minimum evolution, and it presented the evolutionary relationship among different species (Figure S1C). We also found that SETDB1 protein was mainly localized in the nucleoplasm of A-431 (human epithelial carcinoma cell line), U-2 OS (human osteosarcoma cells), and U-251 MG (human brain glioblastoma astrocytoma cancer cells) and vesicles of U-2 OS cell lines (Figure S1D).

### 3.2. Gene Expression Analysis of SETDB1

**Figure 3:**
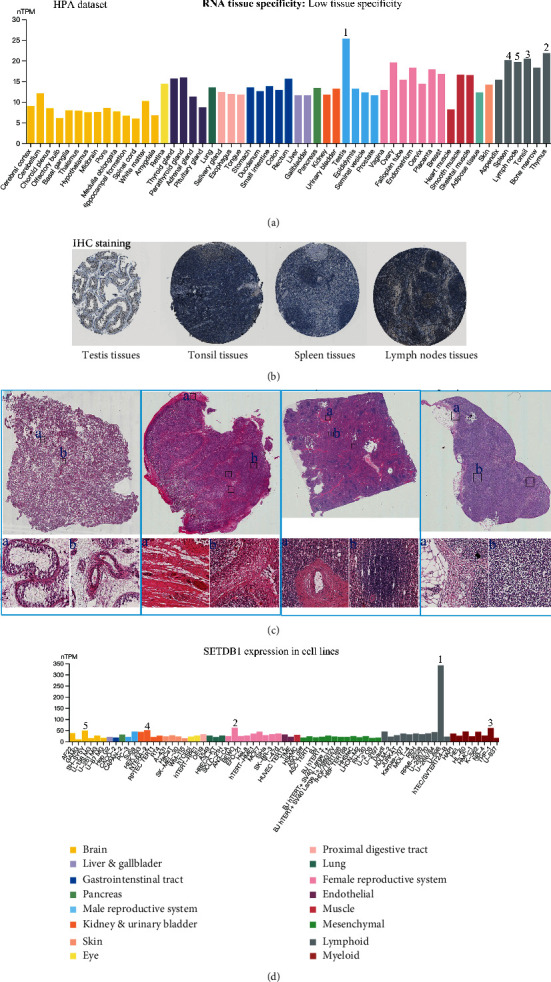
The expression levels of SETDB1 in normal tissues and cell lines. (a) The mRNA expression levels of SETDB1 in normal tissues (data from HPA dataset). (b) Immunohistochemistry results of SETDB1 assessed in testis tissues, tonsil tissues, spleen tissues, and lymph node tissues. (c) Hematoxylin-eosin results of SETDB1 assessed in testis tissues, tonsil tissues, spleen tissues, and lymph node tissues. (d) The mRNA expression levels of SETDB1 in cell lines.

We first confirmed that SETDB1 was widely expressed in human normal and tumor tissues (Figure S2A and Table S1). Then, the SETDB1 mRNA expression levels were compared in nontumor tissues based on the HPA and GTEx database (Figure 3(a) and Figure S2B). The bar charts showed that SETDB1 had the highest expression level in the testis, followed by the thymus, tonsil, spleen, and lymph node, indicating that the SETDB1 was mainly expressed in the bone marrow and lymphoid tissues. The SETDB1 expression level was high in most normal tissues, indicating the low tissue specificity of the SETDB1 mRNA expression. Additionally, the IHC and H&E staining results of the top five normal tissues in terms of SETDB1 expression level were displayed based on HPA online database (Figures 3(b) and 3(c)). No data related to the IHC and H&E staining assessment of SETDB1 in thymus tissues were obtained. The expression levels of SETDB1 in different cell lines were assessed. The results from the HPA database showed that SETDB1 was significantly enriched in U-698, followed by the BEWO, THP-1, NTERA-2, and SH-SY5Y (Figure 3(d)). In addition, SETDB1 single cell specificity is displayed in Figure S2C. The SETDB1 expression level was significantly higher in late spermatids, early spermatids, spermatocytes, oligodendrocytes, and microglial cells. Finally, the SETDB1 expression patterns in testis tissues were assessed using published RNA-sequencing data (Figure S2D).

**Figure 4:**
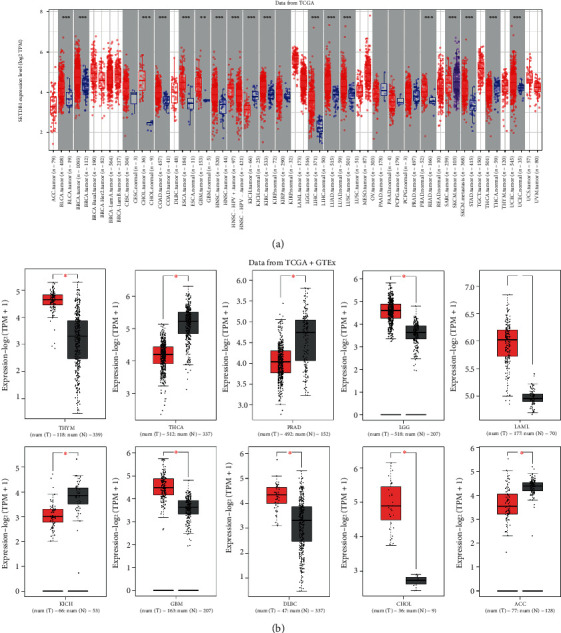
The mRNA expression levels of SETDB1 between cancer and normal tissues. (a) SETDB1 mRNA expression levels in different tumor types and corresponding normal tissue from TCGA datasets. (b) The differences in expression levels of SETDB1 in different tumors and normal tissues from TCGA and GTEx datasets.

In order to further explore the expression levels of SETDB1 in different tumor tissues and paracancerous tissues, we further performed the differential expression analysis using several online databases. In the TIMER database, the SETDB1 expression level was elevated in bladder urothelial carcinoma (BLCA), BRCA, CHOL, colon adenocarcinoma (COAD), esophageal carcinoma (ESCA), GBM, HNSC, KIRC, LIHC, LUAD, lung squamous cell carcinoma (LUSC), rectum adenocarcinoma (READ), stomach adenocarcinoma (STAD), thyroid carcinoma (THCA), and uterine corpus endometrial carcinoma (UCEC) (Figure 4(a)). However, compared with the SETDB1 expression level in paracancerous tissue, that in KICH (kidney chromophobe) was lower (Figure 4(a)). The mRNA expression levels of SETDB1 in HNSC-HPV^+^ tumor and SKCM-Metastasis tissues were higher than those in HNSC-HPV^−^ tumor and SKCM primary tumor tissues (Figure 4(a)). These data are in agreement with the expression levels of SETDB1 in tumor tissues and paracancerous tissues in Sangerbox and UALCAN databases (Figure S3). The expression of SETDB1 in SKCM was lower in patients with primary tumors than that in patients with metastasis tumors, indicating that a high expression level of SETDB1 in SKCM may imply metastasis (Figure 4(a)). In the GEPIA2 online database, combined with the TCGA and GTEx datasets, the differential expression analysis showed that SETDB1 was highly expressed in the thymoma (THYM), lower grade glioma (LGG), acute myeloid leukemia (LAML), GBM, lymphoid neoplasm diffuse large B-cell lymphoma (DLBC), and CHOL, while it was lowly expressed in THCA, prostate adenocarcinoma (PRAD), KICH, and adrenocortical carcinoma (ACC) and was not expressed in other cancers (Figure 4(b)).

**Figure 5:**
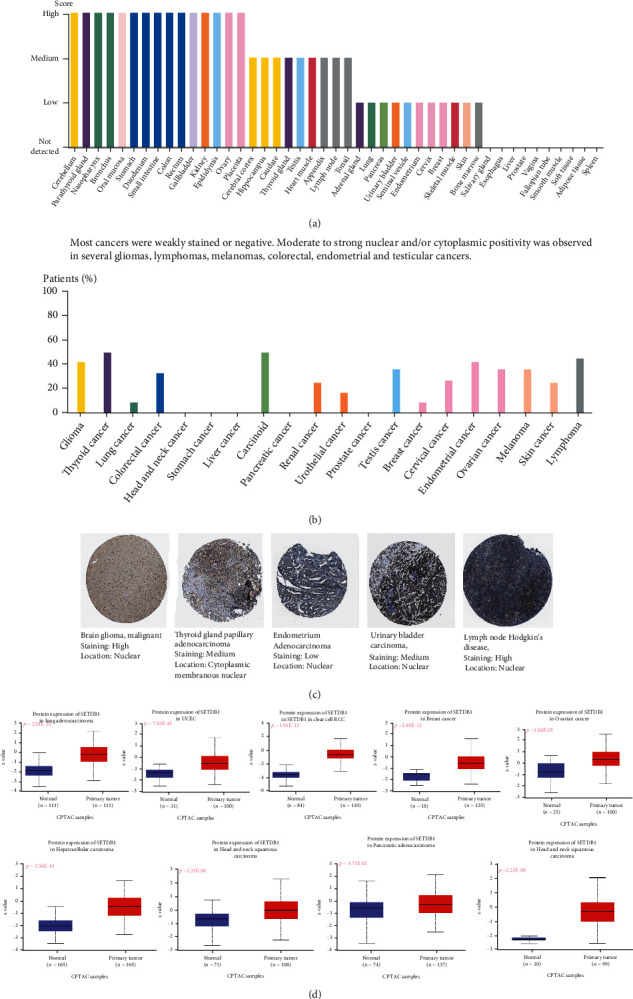
The protein expression levels SETDB1 in normal and cancer tissues. (a) SETDB1 protein expression data in 44 normal tissues. (b) The percentage of cancer patients (maximum 12 patients) with high and medium protein expression level (HPA018142). (c) Immunohistochemistry results of SETDB1 protein assessed in brain glioma, papillary adenocarcinoma, endometrium adenocarcinoma, urinary bladder carcinoma, and lymph node Hodgkin's disease. (d) The differences in protein expression levels of SETDB1 in different tumors and normal tissues from TCGA.

For acquiring more comprehensive expression information, HPA and UALCAN online databases were combined to assess the protein expression of SETDB1 in various cancers and normal tissues. In HPA online dataset, the protein of SETDB1 was observed in 45 normal tissue samples. Among them, 15 samples showed a high expression score, nine samples exhibited a medium expression score, 11 samples exhibited a low expression score, and 10 samples had no expression score (Figure 5(a)). In tumor samples, most cancers were weakly stained or negative. Moderate to strong nuclear and cytoplasmic positivity was observed in several gliomas, lymphomas, melanomas, colorectal cancer, endometrial cancer, and testicular cancer (Figure 5(b)). Furthermore, the classic IHC staining was performed. The results showed that SETDB1 had high expression levels in brain glioma and Hodgkin's lymphoma, had medium levels in THCA and BLCA, and had a low level in endometrium adenocarcinoma (Figure 5(c)). In the “CPTAC” module of UALCAN, we accessed the differences in protein expression of SETDB1 between various cancers and paracancerous tissues. Compared with SETDB1 protein expression levels in the adjacent normal tissues, those in BRCA (*p* = 2.85E − 12), OV (*p* = 1.05E − 05), KIRC (*p* = 7.92E − 45), UCEC (*p* = 1.91E − 12), LUAD (*p* = 2.91E − 31), HNSC (*p* = 2.21E − 08), PAAD (*p* = 3.71E − 02), GBM (*p* = 2.65E − 19), and LIHC (*p* = 1.56E − 41) were significantly higher (Figure 5(d)).

### 3.3. Association of SETDB1 Expression with Clinicopathological Features

**Figure 6:**
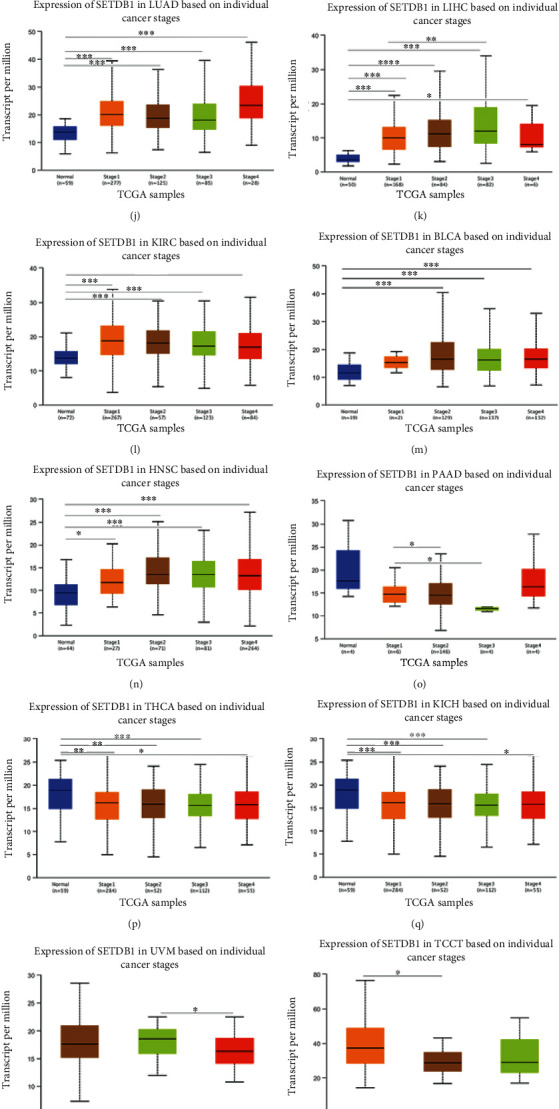
Association analysis of SETDB1 expression with clinicopathological features. (a) The association between SETDB1 expression and pathological clinical stage. (b–s) Correlation between SETDB1 expression and normal as well as pathological clinical stage. (t–w) Correlation between SETDB1 expression and TNM classification (t–v) and clinicopathological grade (w).

GEPIA2 and UALCAN online databases were also applied to assess the association between SETDB1 expression and the clinicopathological stages of various cancers. The results showed that SETDB1 expression was significantly related to clinicopathological stages of tumors, including PAAD (*p* = 0.0444), LIHC (*p* = 0.0132), KICH (*p* = 0.00939), and testicular germ cell tumors (TGCT) (*p* = 0.0468) (Figure 6(a)). Additionally, in the UALCAN online databases, we found that 13 tumors at different clinicopathological stages showed higher SETDB1 expression levels than corresponding normal tissues, and these tumors are BLCA, BRCA, CHOL, COAD, ESCA, HNSC, kidney renal clear cell carcinoma (KIRC), LIHC, LUAD, LUSC, READ, STAD, and UCEC (Figures 6(b)–6(n)). These findings suggested that SETDB1 may be an oncogenic gene in these human malignant tumors. In contrast, the SETDB1 expression level was higher in normal tissues than that in three tumor tissues, namely, KICH, PAAD, and THCA, at clinicopathological stages (Figures 6(o)–6(q)). The results indicate that SETDB1 may be a protective factor in these tumors. Furthermore, for uveal melanoma (UVM) and TGCT patients, the SETDB1 expression levels in cancers at advanced clinicopathological stages were significantly lower than those in tumors at early stages, implying that decreased SETDB1 expression may indicate the tumor progression in these patients (Figures 6(r) and 6(s)). These results demonstrated that abnormal expression of SETDB1 may be associated with the initiation and progression of various human cancers.

We further investigated the association of SETDB1 expression with other clinicopathological features, such as TNM classification and clinicopathological grade based on Sangerbox 3.0. SETDB1 expression in GBM, BRCA, ESCA, SARC (Sarcoma), and PRAD was significantly associated with tumor T classification (Figure 6(t)). SETDB1 expression is significantly associated with N classification in LGG, LIHC, and READ and is related to M classification in LUAD, PAAD, and UVM (Figures 6(u) and 6(v)). Additionally, the clinicopathological grade of LUSC, UCS (uterine carcinosarcoma), and SARC is associated with SETDB1 expression (Figure 6(w)).

### 3.4. Survival Analysis of SETDB1

**Figure 7:**
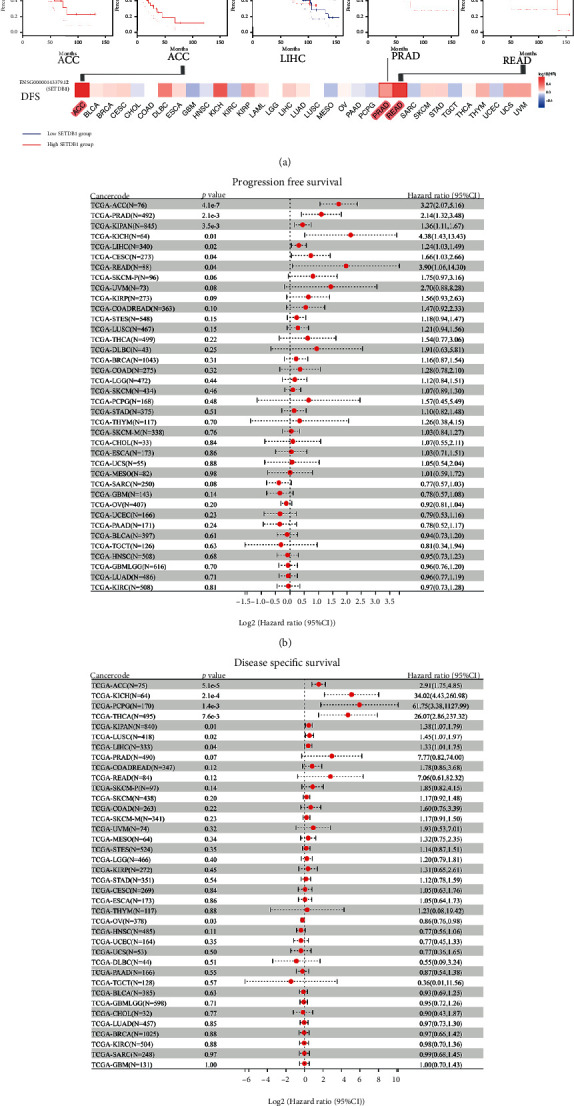
Correlation between SETDB1 expression and survival prognosis of cancers in TCGA datasets. (a) Overall survival. (b) Disease-free survival. (c) Progression-free survival. (d) Disease-specific survival.

To explore the prognostic value of SETDB1 in various human cancers, we classified the cancer samples into high- and low-expression subgroups according to the median expression value of SETDB1. First, the GEPIA2 database was used to perform the OS and DFS analyses in pan-cancer cohorts. In terms of OS, higher expression of SETDB1 was associated with poorer clinical outcomes in ACC (*p* = 0.0055) and LIHC (*p* = 0.0290) (Figure 7(a)). The results also revealed a correlation between high SETDB1 expression levels and poor DFS in ACC (*p* = 3.9e − 05), READ (*p* = 0.0180), and PRAD (*p* = 0.0150) (Figure 7(a)). It is indicated that abnormally expressed SETDB1 may be a prognostic indicator in these tumors. For acquiring a more comprehensive prognostic value of SETDB1, the Cox analyses based on Sangerbox database were performed for DFI and DSS of various cancer samples. The results revealed that SETDB1 expression influenced DFI in patients with ACC (*p* = 4.1e − 7, HR = 3.27, 95% CI 2.07-5.16), PRAD (*p* = 2.1e − 3 2, HR = 2.14, 95% CI 1.32-3.48), KIPAN (*p* = 3.5e − 3, HR = 1.36, 95% CI 1.11-1.67), KICH (*p* = 0.01, HR = 4.38, 95% CI 1.43-13.43), LIHC (*p* = 0.02, HR = 1.24, 95% CI 1.03-1.49), and cervical squamous cell carcinoma and endocervical adenocarcinoma (CESC) (*p* = 0.04, HR = 1.66, 95% CI 1.03-2.66), and READ (*p* = 0.04, HR = 3.90, 95% CI 1.06-14.30) (Figure 7(b)). However, abnormally expressed SETDB1 was associated with DSS for patients with ACC (*p* = 5.1e − 5, HR = 2.91, 95% CI 1.75-4.85), KICH (*p* = 2.1e − 4, HR = 34.02, 95% CI 4.43-260.98), pheochromocytoma and paraganglioma (PCPG) (*p* = 1.4e − 3, HR = 61.75, 95% CI 3.38-1127.99), THCA (*p* = 7.6e − 3, HR = 26.07, 95% CI 2.86-237.32), KIPAN (*p* = 0.01, HR = 1.38, 95% CI 1.07-1.79), LUSC (*p* = 0.02, HR = 1.45, 95% CI 1.07-1.97), and LIHC (*p* = 0.04, HR = 1.33, 95% CI 1.01-1.75) (Figure 7(c)). We focused on the association between SETDB1 expression and the breast cancer, ovarian cancer, lung cancer, and gastric cancer prognosis. In order to evaluate the prognostic abilities of SETDB1 in these cancers, independent clinical factors were selected as the subgroups (Table S2-S6).

### 3.5. Genetic Alteration Analysis of SETDB1

**Figure 8:**
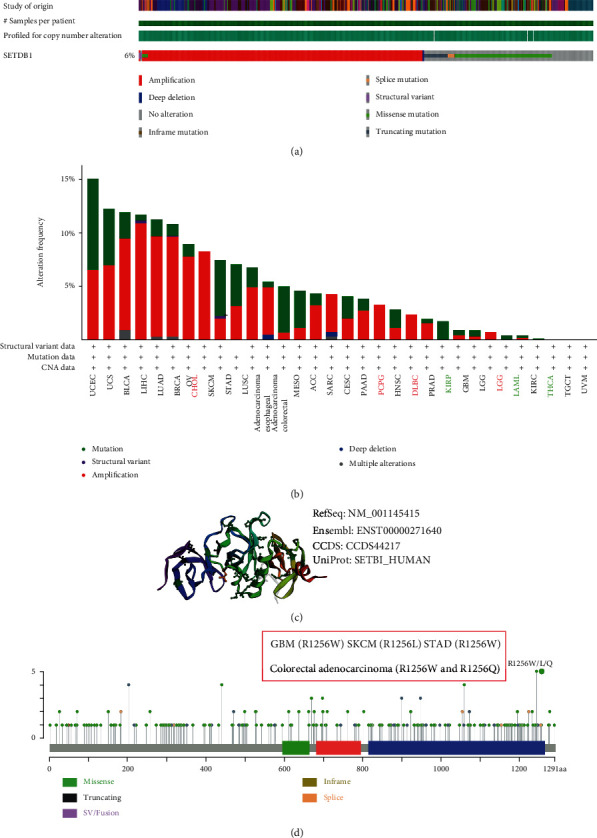
Mutation feature of SETDB1 in different tumors of TCGA. (a) Summary of alterations in SETDB1 expression in different tumors. (b) The alteration frequency with mutation type. (c) Some SETDB1 mutations are shown on the 3D structure of protein. (d) The mutation site of SETDB1 in amino acid sequence.

Malignant tumor is caused by genetic alterations, and mutated genes offer potential molecular therapeutic targets [20, 21]. Given that SETDB1 genetic alterations were associated with molecular therapeutic targets for various human cancers, we investigated the genetic alteration levels of SETDB1 in various human cancers based on TCGA datasets. The results showed that SETDB1 altered 630 cases (6%) out of 10439 cases (data from PanCancer Atlas and TCGA) (Figure 8A). We also found that missense mutation was the main type of SETDB1 mutation, followed by the truncating mutation and splice mutation (Figure 8(a) and Figure S4A). Furthermore, the primary SNV class was C > T (29.81%), followed by G > A (21.12%), A > G (11.47%), and G > T (10.56%) (Figure S4B). By analyzing genetic alterations and expression, we found that SETDB1 genetic alterations induced a switch in the mRNA expression levels of SETDB1 in human tumors. However, few differences were observed in the genetic alteration by deep deletion (Figure S4C). Furthermore, the genetic alteration type in cholangiocarcinoma (CHOL) (8.99% of 523 cases), PCPG (3.37% of 178 cases), DLBC (7.5% of 440 cases), and THYM (0.81% of 123 cases) (Figure 8(b)) was amplification. The genetic alteration type in kidney renal papillary cell carcinoma (KIRP) (1.81% of 276 cases), LAML (0.5% of 200 cases), and THCA (0.2% of 490 cases) (Figure 8(b)) was missense mutation. Additionally, the SETDB1 mutation frequency in patients with mixed endometrial carcinomas was the highest (15.09% of 517 cases), including 8.51% (44 cases) mutation and 6.58% (34 cases) amplification (Figure 8(b)). The structural variant and deep deletion were rare in human cancers and were only identified in five cancers among cancers included in this research, namely, LIHC, BRCA, and SKCM (structural variant) and ESCA and SARC (deep deletion) (Figure 7(b)). We used the “mutation” module of the cBioPortal database to investigate the type and site of SETDB1 mutation (NM_001145415/ENST00000271640) in each sample. The R1256W/L/Q mutation and translation from R (Arginine) to W (Tryptophan) or L (Leucine) or Q (Glutamine) were observed in the SET conserved domain and occurred in one case of GBM (R1256W), one case of skin cutaneous melanoma (SKCM) (R1256L), one case of STAD (R1256W), and two cases of colorectal adenocarcinoma (R1256W and R1256Q). However, the function of R1256W/L/Q mutation remained unknown (Figure 8(d)). The mutation spectrum of SETDB1 was explored by Sangerbox 3.0 (Figure S4D). Finally, the 3D structure of SETDB1 protein and the mutation of the sequence were displayed (Figure 8(c)). However, the R1256W/L/Q mutation was not displayed in the 3D structure of the SETDB1 protein. The CNV pie chart also showed that the heterozygous amplification of CNV was distributed in most cancers, whereas the heterozygous deletion was predominantly distributed in the KICH (Figure S4E). A significant positive correlation was observed between SETDB1 expression and CNV in various cancers (Figure S4F).

### 3.6. Methylation Analysis of SETDB1

**Figure 9:**
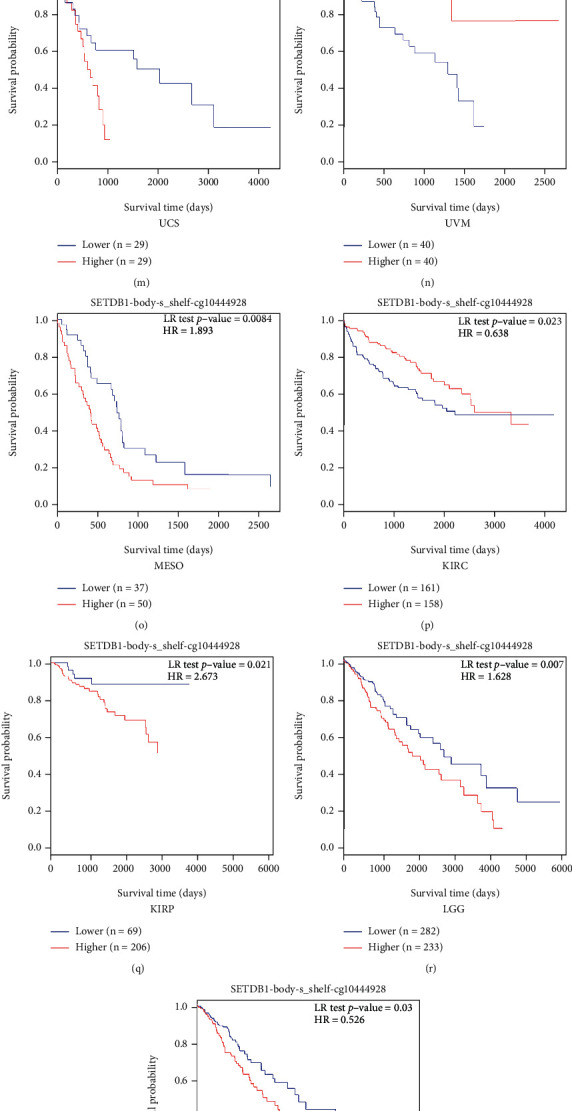
Methylation and protein phosphorylation analysis. (a–l) The differential DNA methylation level of SETDB1 promoter in twelve tumor types. (a) UCEC, (b) TGCT, (c) READ, (d) PRAD, (e) LUSC, (f) LUAD, (g) LIHC, (h) HNSC, (i) ESCA, (j) COAD, (k) BRCA, and (l) BLCA. (m–s) The prognosis value of single CpG (cg10444928) of SETDB1 in seven tumor types. (m) UCS, (n) UVM, (o) MESO, (p) KIRC, (q) KIRP, (r) LGG, and (s) CESC. (t) Phosphorylation site of SETDB1 protein. (u–z) The differential protein level of SETDB1 in S1006 site in six tumor types. (u) Breast cancer, (v) glioblastoma multiforme, (w) pancreatic adenocarcinoma, (x) head and neck squamous carcinoma, (y) lung adenocarcinoma, and (z) clear cell RCC.

Growing evidence showed that aberrant methylation was associated with oncogenesis and may have a significant clinical value [22]. Therefore, we assessed the DNA methylation levels of SETDB1 and its prognosis value in various human cancers. Firstly, we compared the levels of SETDB1 promoter methylation in tumors and paracancerous tissues based on the UALCAN database. The results showed that the promoter methylation levels of SETDB1 in BLCA, BRCA, COAD, ESCA, HNSC, LIHC, LUAD, LUSC, PRAD, READ, TGCT, and UCEC were significantly reduced compared with those in paracancerous tissues (Figures 9(a)–9(l)). Correlation analysis showed that SETDB1 expression was significantly positively correlated with RNA modification-related genes (Figure S5). In the MethSurv online database, we evaluated the DNA methylation level and prognostic value of SETDB1 in various human cancers, and the relative methylation level was displayed in Figure S6. It can be seen that cg10444928 site of SETDB1 in 25 human tumors showed the highest DNA methylation level. To analyze the association of the cg10444928 site of SETDB1 with prognosis across various human cancers, we explored the prognosis value of single CpG (cg10444928) of SETDB1 based on the “single CpG” module of MethSurv database. The results showed that cg10444928 of SETDB1 was significantly associated with the prognosis of UCS (*p* = 1.50E − 02, HR = 2.514), UVM (*p* = 6.30E − 04, HR = 0.196), mesothelioma (MESO) (*p* = 8.40E − 03, HR = 1.893), LGG (*p* = 7.00E − 03, HR = 1.628), KIRP (*p* = 2.10E − 02, HR = 2.673), KIRC (*p* = 2.30E − 02, HR = 0.638), and CESC (*p* = 0.03, HR = 0.526) (Figures 9(m)–9(s)). The prognostic value of other single CpGs of SETDB1 in 25 cancers was also assessed using the “all cancers” module of the MethSurv database (Table S7).

### 3.7. Protein Phosphorylation Analysis of SETDB1

Protein phosphorylation may be a promoter or a suppressor of oncogenesis. Therefore, exploring protein phosphorylation is beneficial to developing a novel antitumor agent in human tumors [23]. We first explored the protein phosphorylation site of SETDB1 based on the PhosphoSitePlus database. As shown in Figure 9(t), the most predominant protein phosphorylation locus for the SETDB1 is Ser1006 (flanking sequence: RNYGYNPsPVkPEGL) located in the SET conserved domain. Subsequently, we assessed the differences in phosphorylation levels at the single phosphorylation site of SETDB1 between tumor tissues and paracancerous tissues using the CPTAC dataset. The Ser1006 locus of SETDB1 possessed a higher phosphorylation level in BRCA (*p* = 1.55E − 08), GBM (*p* = 1.27E − 02), PAAD (*p* = 1.00E − 11), HNSC (*p* = 1.16E − 33), LUAD (*p* = 1.91E − 32), and KIRC (*p* = 8.36E − 17) (Figures 9(u)–9(z)). These results implied that protein phosphorylation of SETDB1 at Ser1006 locus may play an important role in the development and progression of those tumors. Our previous findings suggested that SETDB1 protein was mainly located in the nucleoplasm. However, whether the protein phosphorylation of SETDB1 at Ser1006 locus affects its location or its function remains unknown and requires more investigations.

### 3.8. Immune and Molecular Subtype Analysis of SETDB1

**Figure 10:**
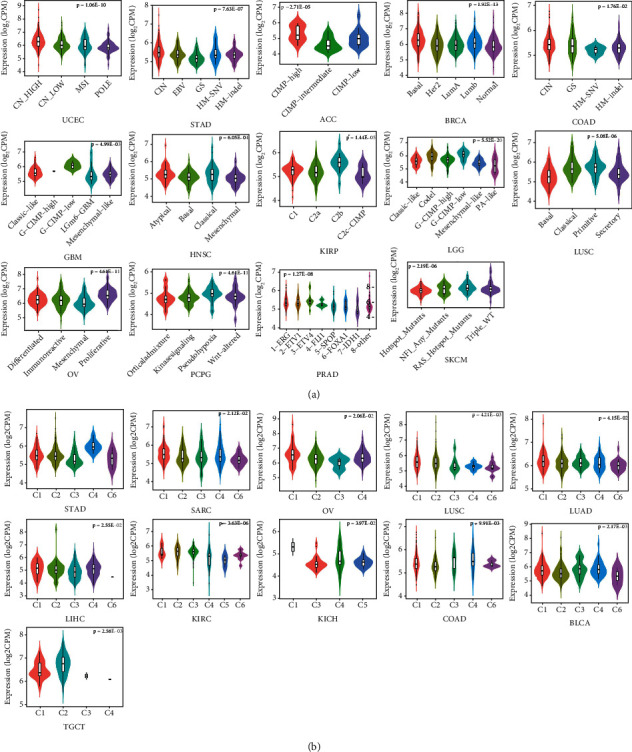
The immune and Molecular Subtypes analysis of SETDB1. (a) The SETDB1 gene differential expression in different immune subtypes. (b) The SETDB1 gene differential expression in different molecular subtypes.

We assessed the relationship between SETDB1 expression status and immune activity as well as molecular subtypes in human cancers based on the TISIDB database. According to immune activity, the tumor tissues were divided into C1 (wound healing), C2 (IFN-gamma dominant), C3 (inflammatory), C4 (lymphocyte depleted), C5 (immunologically quiet), and C6 (TGF-b dominant). In order to verify the dynamic relationship between SETDB1 expression status and immune activity, we assessed the immune activity levels of the six subtypes in various cancers. The results showed that SETDB1 expression was significantly associated with immune subtypes in BLCA, COAD, KICH, KIRC, LIHC, LUAD, LUSC, ovarian serous cystadenocarcinoma (OV), SARC, STAD, and TGCT (Figure 10(a)). Similarly, the SETDB1 expression was associated with molecular subtypes in ACC, BRCA, COAD, GBM, HNSC, KIRP, LGG, LUSC OV, PCPG, PRAD, SKCM, STAD, and UCEC (Figure 10(b)). These results implied that SETDB1 expression status was relevant to the immune subtypes and molecular subtypes of various cancers.

### 3.9. Immune Infiltration Analysis of SETDB1

**Figure 11:**
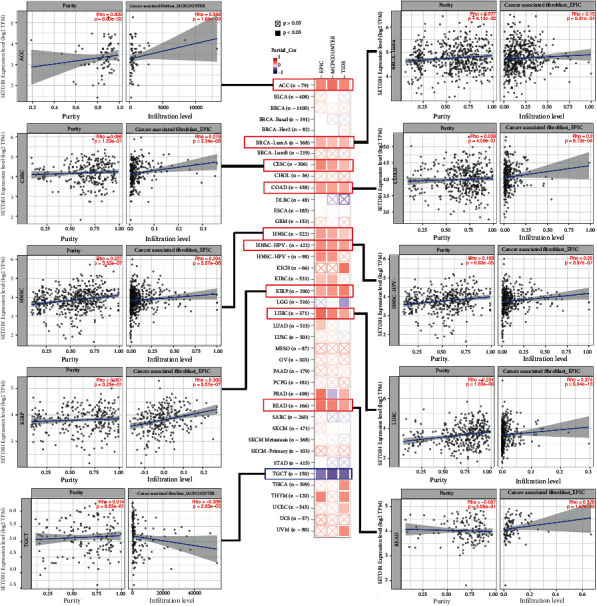
Correlation analysis between SETDB1 expression and immune infiltration of cancer-associated fibroblasts.

Considering the importance of the immune microenvironment in tumorigenesis and cancer progression, we characterized immune infiltration levels of SETDB1 based on several databases. CAFs are the fibroblasts around tumor cells and the major stromal cells in the tumor microenvironment. They play a significant role in the initiation and progression of tumors [24, 25]. It has been demonstrated that targeting CAFs is an effective treatment strategy for various cancers [26]. The EPIC, MCPCOUNTER, and TIDE algorithms were applied to assess the relationship between the infiltration level of CAFs and SETDB1 gene expression in various human cancers. We observed a significantly positive correlation between SETDB1 expression and infiltration level of CAFs in ACC (Rho = 0.358, *p* = 1.88e − 03), BRCA (Rho = 0.15, *p* = 6.31e − 04), CESC (Rho = 0.279, *p* = 2.34e − 06), COAD (Rho = 0.2, *p* = 8.73e − 04), HNSC (Rho = 0.204, *p* = 5.07e − 06), HNSC-HPV (Rho = 0.25, *p* = 3.97e − 07), KIRP (Rho = 0.306, *p* = 5.57e − 07), LIHC (Rho = 0.374, *p* = 6.64e − 13), and READ (Rho = 0.329, *p* = 1.57e − 03) but a strongly negative correlation in TGCT (Rho = −0.339, *p* = 2.63e − 05) (Figure 11). Furthermore, partial correlation analysis between SETDB1 expression and immune cell infiltration was conducted using the TIMER2.0 database. The results demonstrated a remarkable correlation between SETDB1 expression and CD8^+^ T cells, CD4^+^ T cells, Tregs, and B cells (Figure S7A-7D).

### 3.10. Correlation Analysis of Immune Checkpoint Inhibitor-Related Genes

**Figure 12:**
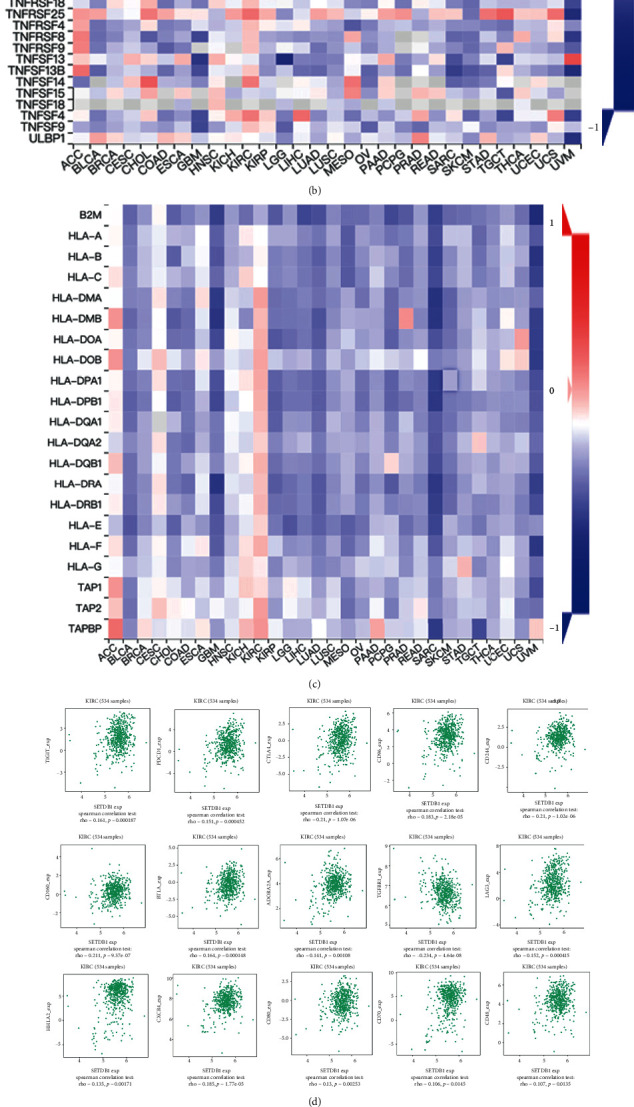
Correlation between SETDB1 expression and immunoinhibitor, immunostimulatory, human leukocyte antigen- (HLA-) associated genes, microsatellite instability, and tumor mutational burden. (a) Immunoinhibitor, (b) immunostimulator, and (c) human leukocyte antigen- (HLA-) associated genes. Red color indicates positive correlations; blue color indicates negative correlations. (d) SETDB1 expression significantly correlated with immunoinhibitor and immunostimulator in KIRC tissues. (e) Microsatellite instability and (f) tumor mutational burden.

Accumulating evidence suggests that immune checkpoint inhibitors are a class of biologics that interact with the immune system to encourage antitumor response by immune cells [27]. We also demonstrated a significant correlation between SETDB1 expression and each immune checkpoint-related gene (immunoinhibitor and immunostimulator) in diverse cancers in TCGA (Figures 12(a) and 12(b)). For example, in KIRC, SETDB1 expression has a significantly positive correlation with the expression of TIGIT, PDCD1, CTLA4, CD96, CD244, CD160, BTLA, ADORA2A, TGFBR1, LAG3, HHLA2, CXCR4, CD80, CD70, CD48, etc. (Figure 12(d)). The MHC is a human leukocyte antigen (HLA) that plays an important role in tumor immunotherapy by activating T cells [28, 29]. According to our results, the association between SETDB1 expression and HLA-related genes varies markedly among cancer types. The SETDB1 expression and HLA-related genes in ACC, CESC, and KIRC were positively correlated but were negatively correlated in other cancers in TCGA (Figure 12(c)). TMB and MSI are emerging predictors associated with survival and response to immunotherapy [30, 31]. This study showed that SETDB1 was positively correlated with MSI in BLCA, CESC, LUAD, LUSC, READ, and SARC, while negatively correlated with MSI in DLBC, but did not show correlation with MSI in other cancers (Figure 12(e)). SETDB1 expression was positively correlated with TMB in BLCA, BRCA, LGG, LUAD, and STAD, while negatively correlated with TMB in THCA and UCS, but did not correlate with TMB in other cancers (Figure 12(f)).

### 3.11. Function and Pathway Analysis of SETDB1-Related Genes

**Figure 13:**
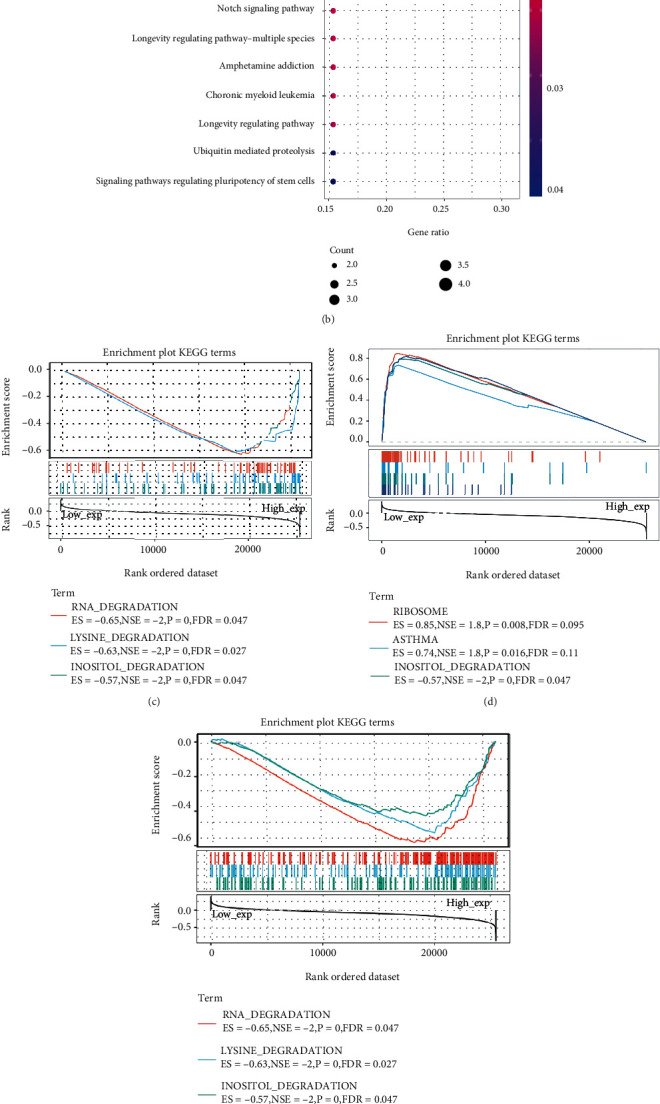
Gene Ontology (GO) functional enrichment and Kyoto Encyclopedia of Genes and Genomes (KEGG) pathway and Gene Set Enrichment Analysis (GSEA) for SETDB1 and its related genes. (a) GO analysis for SETDB1-related genes. (b) KEGG analysis for SETDB1-related genes. (c) The enriched gene sets in KEGG collection by the high SETDB1expression sample. (d) The enriched gene sets in KEGG by samples with low SETDB1 expression. (e) Enriched gene sets in HALLMARK by samples of high SETDB1 expression. (f) Enriched gene sets in HALLMARK by the low SETDB1 expression.

To further elucidate the biological function and molecular mechanism of SETDB1 and provide theoretical support for the study of tumorigenesis, we identified the targeting SETDB1-binding proteins with the STRING tool and conducted bioinformatics analyses. The interaction network analysis showed interaction information of SETDB1 and these proteins with 51 nodes and 715 edges (Figure S8A). These genes were considered the target genes to obtain enriched GO terms and significant KEGG pathways. In GO analysis, 364 GO categories were detected, including 251 biological process (BP), 40 cellular component (CC), and 73 molecular function (MF). In the GO-BP category, the target genes were mainly enriched in covalent chromatin modification (GO:0016569), histone modification (GO:0016570), and peptidyl-lysine modification (GO:0018205) (Figure 13(a)). In the GO-CC category, the genes were related to heterochromatin (GO:0000792), chromosomal region (GO:0098687), chromosome, and telomeric region (GO:0000781) (Figure 13(a)). In the GO-MF category, target genes were mainly enriched in transcription coregulator activity (GO:0003712), methylated histone binding (GO:0035064), and methylation-dependent protein binding (GO:0140034) (Figure 13(a)). In KEGG pathway analysis, 51 genes were categorized into 11 KEGG pathways. As a result, lysine degradation, thyroid hormone signaling pathway, and cell cycle were identified and marked as main KEGG pathways (Figure 13(b)). These results are consistent with the results of GSEA analysis (Figures 13(c) and 13(d)). Additionally, in terms of the HALLMARK, a high expression level of SETDB1 was significantly enriched in the mitotic spindle, unfolded protein response, and PI3K-AKT-MTOR signaling. In contrast, the low expression level of SETDB1 was significantly enriched in the inflammatory response, allograft rejection, coagulation, and epithelial-mesenchymal transition (Figures 13(e) and 13(f)). Furthermore, the results of GeneMANIA also revealed that SETDB1 and targeting SETDB1-binding proteins were mainly related to chromatin assembly, chromatin assembly or disassembly, DNA packaging, DNA conformation change, protein-DNA complex, nucleosome organization, and DNA packaging complex (Figure S8B). CeRNA is important for tumorigenesis by forming an extensive ceRNA network involving mRNA, miRNA, and ncRNA. We identified hsa-miR-29a-3p as the most vital miRNA regulator by overlapping predictions of three databases (Figure S8C). We then explored the complementary sequences between SETDB1 and hsa-miR-29a-3p using the TargetScanHuman database (Figure S8D). We also predicted the twelve target lncRNAs by interacting with miRNA and lncRNA sequences in the LncBase database. Then, the lncRNA-miRNA-mRNA network was constructed based on lncRNA-miRNA and miRNA-mRNA regulation pairs (Figure S8E).

### 3.12. Drug Sensitivity Analysis

**Figure 14:**
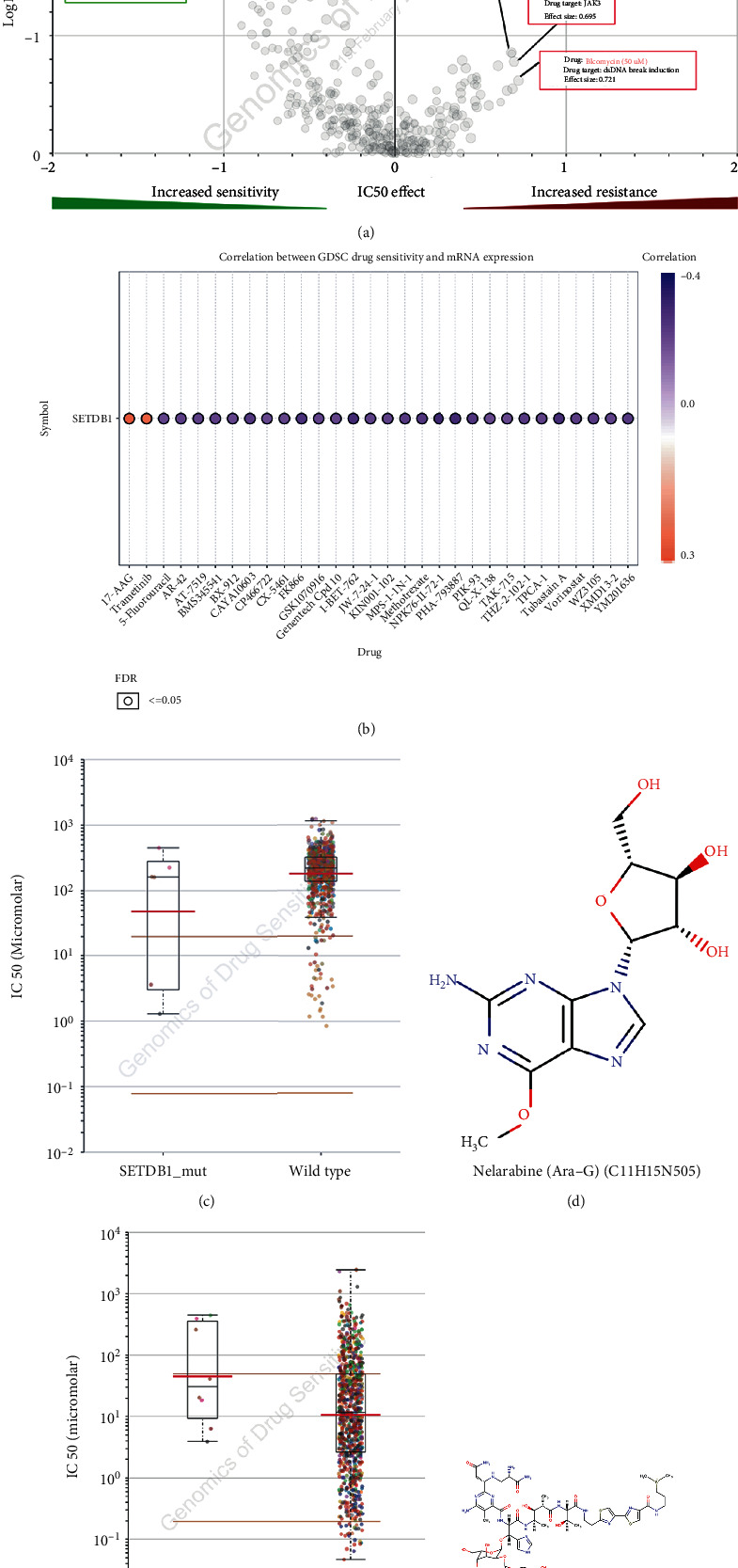
Drug sensitivity analysis based on SETDB1 mutation. (a) Genomics of drug sensitivity in cancer. (b) Correlation between GDSC drug sensitivity, drug resistance, and SETDB1 mutation. (c) Nelarabine (Ara-G) IC50 values for SETDB1 mutation. (d) The structural formulas of Nelarabine (Ara-G). (e) Bleomycin IC50 values for SETDB1 mutation. (f) The structural formulas of Bleomycin.

Genetic mutations can influence the efficacy of chemotherapy and targeted therapy. Therefore, we evaluated the role of SETDB1 in chemotherapy or targeted therapy and investigated the drug sensitivity and drug resistance of cancer cell lines from the GDSC datasets. ANOVA analysis showed that drug sensitivity toward Arg-G (nelarabine), Nilotinib, and KIN001-042 was significantly correlated with the expression of SETDB1 (negative correlation with IC50). However, the drug resistance toward Bleomycin (50 *μ*M), JAK3_7406, and FGFR_0939 was correlated with the expression of SETDB1 (positive correlation with IC50) (Figure 14(a)). The correlation between GDSC drug sensitivity and SETDB1 expression showed that most drugs were negatively correlated with SETDB1 expression, while 17-AAG and trametinib showed a positive correlation with SETDB1 expression (Figure 14(b)). Furthermore, the IC50 values of nelarabine and Bleomycin (50*μ*M) for SETDB1 (Mut and wild type) were displayed. The chemical formulas of nelarabine and Bleomycin are C_11_H_15_N_5_O_5_ and C_55_H_84_N_17_O_21_S_3_, respectively, and their structural formulas are shown in Figures 14(c) and 14(d).

### 3.13. Validating Expression of SETDB1 by IHC

**Figure 15:**
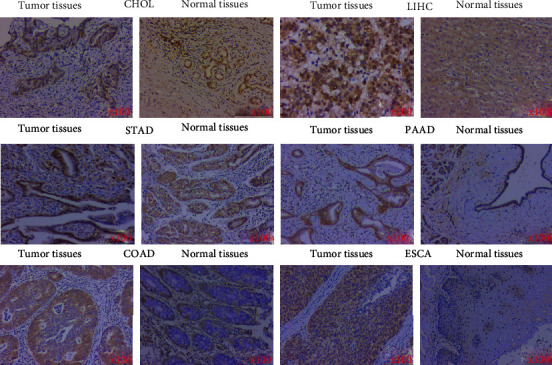
Immunohistochemistry results of SETDB1 performed in normal and tumor tissues of human.

For further validation, the expression level of SETDB1 gene was analyzed by IHC. Since gastrointestinal malignancies are the most frequent primary tumors, we focus on the expression level of SETDB1 gene in the most common gastrointestinal malignancies including LIHC, CHOL, COAD, ESCA, PAAD, and STAD. The results showed that SETDB1 was highly expressed in tumor tissues and rarely expressed in normal tissues (Figure 15). These results are similar to those we have previously reported for different database.

## 4. Discussion

The SETDB1 gene, first identified in 1999 by Harte et al. [11], with a length of 38.6 kilobases (kb), is located on the human chromosome 1q21.3. Chromosome numbers and structural abnormalities are important factors for tumorigenesis and the therapeutic response [32, 33]. Tumor tissues have many chromosomal variants. Chromosome 1q gains occurred in various human cancers, such as LUAD, LIHC, OV, BRCA, and multiple myeloma [32, 34, 35]. Chromosome 1q21.3 abnormalities are related to breast cancer recurrence, and they can promote cell proliferation and DNA damage response in metastatic melanoma [35, 36]. SETDB1 is located in the 1q21.3 region that encodes a histone methyltransferase which regulates transcriptional repression, histone methylation, and gene silencing [37, 38]. This study has demonstrated that the SETDB1 is differentially expressed in most tumors and normal tissues, indicating that it also plays an oncogenic role in these tumors. The amplification of SETDB1 in human tumors is significantly associated with immune exclusion and tumor progression, but its biological and functional role or contribution to tumor prognosis is unknown [12]. This paper is the first pan-cancer analysis of SETDB1 across 33 different tumors based on the data of TCGA, CPTAC, and GEO databases. The results show that SETDB1 is significantly correlated with tumorigenesis and clinical outcomes.

SETDB1 has specific domains [39], such as two Tudors, MBD, pre-SET, SET, and post-SET, and this result is consistent with our finding. The most biological function of SETDB1 is ascribed to the SET domain, which is highly conserved across species and originally identified in the Drosophila Trithorax (TRX) and human MLL proteins [40]. SETDB1 is a member of SET family and is an H3K9 methyltransferase that modulates gene activity. The pre-SET, SET, and post-SET domains are crucial for histone methyltransferase activity. Furthermore, SETDB1 protein has a canonical CpG DNA methyl binding domain (MBD) at the N-terminus, which can bind methylated DNA at one site [41]. Growing evidence suggests the involvement of MBD genes in cancers [42]. MBD is involved in various signaling pathways and cellular functions, including DNA damage repair, chromatin remodeling, histone methylation, and X chromosome inactivation [42]. MBD can also potentially coordinate the functions of DNA methyl-CpG binding and H3K9 methylation, both of which can promote epigenetic marks [42, 43]. SETDB1 also contains a unique tandem Tudor domain that recognizes histone H3 sequences containing acetylated lysines and methylated [44]. SETDB1 biological function is a two-edged sword. On the one hand, it may downregulate antioncogenes through histone methylation. On the other hand, it may inhibit tumor-intrinsic immunogenicity, enabling cancer cells to evade immune responses [12, 45].

A recent study indicated that tumor cell-intrinsic epigenetic alterations drive tumorigenesis and cancer progression [46]. The epigenetic characters reflect the heterogeneity of tumors and indicate potential epigenetic changes, which lead to cancer cell invasion during tumor progress [46, 47]. As an important player in tumor epigenetics, SETDB1 expression is significantly differential in most cancerous tissues and adjacent healthy tissues [8, 48–51], which is consistent with our findings. It is demonstrated that SETDB1 is an oncogene and an important prognostic factor in some tumors. SETDB1 expression is upregulated in LIHC tissues and is associated with tumor size, enhanced stage, and TNM classification [52]. Similarly, for TCGA-LIHC patients, we observed that the expression level of SETDB1 is significantly elevated in tumor tissues compared to that in paracancerous tissues. LIHC tissues from patients with advanced-stage tumors show significantly higher expression levels of SETDB1 compared with those from patients with early-stage tumors. We also observed that the expression levels of SETDB1 were significantly lower in the stage 4 tumor than those in early-stage tumors. However, this result may be inaccurate due to the sample size limitation (six samples with stage 4 tumor). Therefore, a large sample size study is needed to further verify the conclusion.

Cancer develops as a result of genetic mutational events that lead either to the overexpression of growth-promoting oncogenes or the inactivation of cell cycle-controlling tumor suppressor genes [53]. Growing evidence implies that SETDB1 is a potential oncogene for tumorigenesis [8]. Therefore, comprehensively understanding the biological functions of SETDB1 mutations can help to inhibit tumorigenesis and develop effective antitumor agents. It has been reported that mutated SETDB1 is widespread and occurs in most malignant pleural mesothelioma [54, 55]. The frequent SETDB1 mutation indicates that there may be a potential therapeutic target for malignant pleural mesothelioma [55]. We first used the cBioPortal online database to explore genetic mutation levels of SETDB1 in various cancers. The pan-cancer mutation spectra showed that a high mutant frequency of SETDB1 occurred in most human tumors, with the highest frequency in UCEC (15.09% of 517 cases). These results also demonstrated that SETDB1 mutation played a significant role in tumorigenesis.

Cancer is an increasingly health-threatening disease that has a poor prognosis due to the lack of effective treatment. The progression and recurrence of the tumor challenge the effectiveness of therapies [56, 57]. Due to the therapeutic resistance and tumor relapse after therapy, the paradigms of cancer-centric therapeutics are not sufficient to eradicate the malignancy [58]. Targeting tumor microenvironment (TME) is a novel tumor treatment strategy in recent years. CAFs are the most abundant stromal cells in the TME and play significant roles in tumor development. Our results also revealed the significant association between SETDB1 expression and tumor-related immune cell infiltration level of CAFs in certain tumors, including ACC, BRCA, CESC, COAD, HNSC, HNSC-HPV, KIRP, LIHC, READ, and TGCT. Furthermore, we used the online databases to explore the correlation between SETDB1 expression and immune cell infiltration level in human cancer and found that tumor-related immune cells significantly increased in tumor tissues with high SETDB1 expression levels. These results also demonstrated that the expression levels of SETDB1 influenced tumor growth, metastasis, and prognosis.

Immunotherapy has emerged as a new pillar of cancer treatment in recent years. The introduction of PD-1, PD-L1, and CAR-T cell immunotherapy into the therapeutic strategy of advanced cancer leads to unprecedentedly prolonged survival for patients [59]. According to our findings, increased expression of SETDB1 has a significantly negative correlation with immunoinhibitor and immunostimulator in most cancers. Therefore, we speculated that decreasing SETDB1 expression in tumor cells might enhance immunotherapeutic responses.

## 5. Conclusion

In summary, we conducted the pan-cancer analysis of SETDB1 oncogenes for the first time. The omics analysis, prognostic analysis, methylation and phosphorylation analysis, immune analysis, and enrichment analysis of SETDB1 were performed. The mRNA and protein expression levels and gene alteration levels were analyzed. It is expected that the investigation and characterization of SETDB1 biological function can help to identify the key targets and regulatory pathways and promote human cancer treatment in the future.

## Figures and Tables

**Table 1:** The mRNA and protein information of SETDB1.

mRNA Accession number	Protein Accession number	Protein isoform	Conserved domains		
			Superfamily	Location	Conserved domain
NM_001145415.2 NM_001393960.1	NP_001138887.1 NP_001380889.1	Histone-lysine N-methyltransferase SETDB1 isoform 1	smart00391	597 → 672	MBD
			cd10517	674 → 875	SET_SETDB1
			cl40432	1202 → 1291	SET
			pfam18300	193 → 250	DUF5604
			cd20382	260 → 341	Tudor_SETDB1_rpt1
			cd21181	348 → 401	Tudor_SETDB1_rpt2
NM_012432.4	NP_036564.3	Histone-lysine N-methyltransferase SETDB1 isoform 2	smart00391	597 → 672	MBD
			smart00468	679 → 786	PreSET
			smart00333	348 → 400	TUDOR
			smart00317	1206 → 1267	SET
NM_001243491.2 NM_001393964.1 NM_001393965.1 NM_001393966.1	NP_001230420.1 NP_001380893.1 NP_001380894.1 NP_001380895.1	Histone-lysine N-methyltransferase SETDB1 isoform 3	pfam18300	193 → 250	DUF5604
			cd20382	260 → 341	Tudor_SETDB1_rpt1
			cl02573	348 → 380	Tudor_SF
NM_001366417.1 NM_001366418.1 NM_001393958.1 NM_001393959.1	NP_001353346.1 NP_001353347.1 NP_001380887.1 NP_001380888.1	Histone-lysine N-methyltransferase SETDB1 isoform 4	smart00391	598 → 673	MBD
			cd10517	675 → 876	SET_SETDB1
			cl40432	1203 → 1292	SET
			pfam18300	193 → 250	DUF5604
			cd20382	260 → 341	Tudor_SETDB1_rpt1
			cd21181	348 → 401	Tudor_SETDB1_rpt2
NM_001393961.1	NP_001380890.1	Histone-lysine N-methyltransferase SETDB1 isoform 5	smart00391	598 → 673	MBD
			smart00333	348 → 400	TUDOR
			cd10517	675 → 876	SET_SETDB1
			cl40432	1203 → 1291	SET
NM_001393967.1 NM_001393968.1	NP_001380896.1 NP_001380897.1	Histone-lysine N-methyltransferase SETDB1 isoform 6	pfam18300	193 → 225	DUF5604

MBD: methyl-CpG binding domain; SET_SETDB1: SET domain (including pre-SET and post-SET domains) found in SET domain bifurcated 1 (SETDB1) and similar proteins; SET: SET (Su(var)3-9, Enhancer-of-zeste, Trithorax) domain superfamily; DUF5604: domain of unknown function (DUF5604); Tudor_SETDB1_rpt1: first Tudor domain found in SET domain bifurcated 1 (SETDB1) and similar proteins; Tudor_SETDB1_rpt2: second Tudor domain found in SET domain bifurcated 1 (SETDB1) and similar proteins; TUDOR: Tudor domain; Tudor_SF: Tudor domain superfamily.

## Data Availability

The data comes from the public database including TCGA, GEO, GTEx, and CPTAC.

## Abbreviations

SETDB1: SET domain bifurcated histone lysine methyltransferase 1
ACC: Adrenocortical carcinoma
BLCA: Bladder urothelial carcinoma
CESC: Cervical squamous cell carcinoma and endocervical adenocarcinoma
ESCA: Esophageal carcinoma
HNSC: Head and neck squamous cell carcinoma
KIRC: Kidney renal clear cell carcinoma
KIRP: Kidney renal papillary cell carcinoma
LAML: Acute myeloid leukemia
LGG: Lower grade glioma
LIHC: Liver hepatocellular carcinoma
MESO: Mesothelioma
PAAD: Pancreatic adenocarcinoma
READ: Rectum adenocarcinoma
SARC: Sarcoma
SKCM: Skin cutaneous melanoma
UCE: Uterine corpus endometrial carcinoma
UCS: Uterine carcinosarcoma
UVM: Uveal melanoma
CHOL: Cholangiocarcinoma
COAD: Colon adenocarcinoma
GBM: Glioblastoma multiforme
KICH: Kidney chromophobe
LUAD: Lung adenocarcinoma
LUSC: Lung squamous cell carcinoma
DLBC: Lymphoid neoplasm diffuse large B-cell lymphoma
OV: Ovarian serous cystadenocarcinoma
PCPG: Pheochromocytoma and paraganglioma
PRAD: Prostate adenocarcinoma
STAD: Stomach adenocarcinoma
TGCT: Testicular germ cell tumors
THYM: Thymoma THCA thyroid carcinoma
K-M: Kaplan-Meier
OS: Overall survival
H3K9: Histone H3 lysine 9
CDS: Coding sequence
NCBI: National Center for Biotechnology Information
IBS: Illustrator for Biological Sequences
HPA: Human Protein Atlas
UCSC: University of California, Santa Cruz
TCGA: The Cancer Genome Atlas
IHC: Immunohistochemical
H&: Hematoxylin-eosin
DFS: Disease-free survival
DSS: Disease-specific survival
PFI: Progression-free interval
GEO: Gene Expression Omnibus
CNV: Copy number variation
SNV: Single nucleotide variation
COSMIC: Catalogue Of Somatic Mutations In Cancer
TMB: Tumor mutational burden
MSI: Microsatellite instability
GO: Gene Ontology
KEGG: Kyoto Encyclopedia of Genes and Genomes.

## Supplementary Materials

Supplementary Materials The supplementary material for this article can be found at the module of Supplementary figure and table legends. Figure S1: SETDB1 protein, location, and conservation analysis. (A) The main location of SETDB1 protein in cells. (B) Comparison of protein sequences encoded by SETDB1 among ten different species. (C) The phylogenetic tree of SETDB1 in different species. (D) The sites of SETDB1 protein in the U-251 MG cell, U-2 OS cells, and A-431 cells. Figure S2: the expression levels of SETDB1 in normal and tumor tissues as well as different single cell types. (A) The expression levels of SETDB1 in normal and tumor tissues in human body. (B) The mRNA expression levels of SETDB1 in normal tissues (data from GTEx and release V6). (C) The mRNA expression levels of SETDB1 in different single cell types. (D) The SETDB1 expression patterns in testis tissues (data from published RNA-sequencing information). Figure S3: the differential expression level of SETDB1 between normal tissues and tumor tissues. (A) Data from TCGA+GTEx. (B) Data from TCGA. Figure S4: SETDB1 mutation analysis. (A) The main mutation type of SETDB1. (B) The primary single nucleotide variation (SNV) class type of SETDB1. (C) Correlation between SETDB1 expression and different mutated types. (D) The mutation spectrum of SETDB1. (E) The copy number variation (CNV) percentage of SETDB1 in each cancer. (F) Correlation between SETDB1 expression and CNV. Figure S5: correlation between SETDB1 expression and RNA modifications related genes. (A) m6A, (B) m1A, and (C) m5C. Figure S6: the heatmap of DNA methylation level of SETDB1 in different cancers. High-expression (red) and low-expression (blue). Figure S7: correlation of SETDB1 expression with immune infiltration level. Figure S8: protein-protein interaction (PPI) network and mRNA-miRNA-lncRNA network. (A) PPI network for SETDB1 and SETDB1-binding proteins using STRING. (B) The PPI network of SETDB1 and SETDB1-binding proteins using GeneMANIA. (C) The result of predicted miRNAs using 3 different databases based on Venn. (D) mRNA-miRNA-lncRNA network for SETDB1. Table S1: the SETDB1 expression level in normal tissues and tumor tissues based on the GEPIA2. Table S2: subgroup analysis on the correlation of SETDB1 expression and prognosis of gastric cancer cases. Table S3: subgroup analysis on the correlation of SETDB1 expression and prognosis of liver cancer cases. Table S4: subgroup analysis on the correlation of SETDB1 expression and prognosis of lung cancer cases. Table S5: subgroup analysis on the correlation of SETDB1 expression and prognosis of ovarian cancer cases. Table S6: subgroup analysis on the correlation of SETDB1 expression and prognosis of breast cancer cases.
