# Supplementary material for: Pancancer Analyses Reveal Genomics and Clinical Characteristics of the SETDB1 in Human Tumors
Source: J Oncol. 2022 May 23;2022:6115878. doi: 10.1155/2022/6115878 (PMC9152430; doi:10.1155/2022/6115878)
Supplement: Supplementary Materials — The supplementary material for this article can be found at the module of Supplementary figure and table legends. Figure S1: SETDB1 protein, location, and conservation analysis. (A) The main location of SETDB1 protein in cells. (B) Comparison of protein sequences encoded by SETDB1 among ten different species. (C) The phylogenetic tree of SETDB1 in different species. (D) The sites of SETDB1 protein in the U-251 MG cell, U-2 OS cells, and A-431 cells. Figure S2: the expression levels of SETDB1 in normal and tumor tissues as well as different single cell types. (A) The expression levels of SETDB1 in normal and tumor tissues in human body. (B) The mRNA expression levels of SETDB1 in normal tissues (data from GTEx and release V6). (C) The mRNA expression levels of SETDB1 in different single cell types. (D) The SETDB1 expression patterns in testis tissues (data from published RNA-sequencing information). Figure S3: the differential expression level of SETDB1 between normal tissues and tumor tissues. (A) Data from TCGA+GTEx. (B) Data from TCGA. Figure S4: SETDB1 mutation analysis. (A) The main mutation type of SETDB1. (B) The primary single nucleotide variation (SNV) class type of SETDB1. (C) Correlation between SETDB1 expression and different mutated types. (D) The mutation spectrum of SETDB1. (E) The copy number variation (CNV) percentage of SETDB1 in each cancer. (F) Correlation between SETDB1 expression and CNV. Figure S5: correlation between SETDB1 expression and RNA modifications related genes. (A) m6A, (B) m1A, and (C) m5C. Figure S6: the heatmap of DNA methylation level of SETDB1 in different cancers. High-expression (red) and low-expression (blue). Figure S7: correlation of SETDB1 expression with immune infiltration level. Figure S8: protein-protein interaction (PPI) network and mRNA-miRNA-lncRNA network. (A) PPI network for SETDB1 and SETDB1-binding proteins using STRING. (B) The PPI network of SETDB1 and SETDB1-binding proteins using GeneMANIA. (C) The result of [file 6115878.f1.zip › Figure S1.pdf]

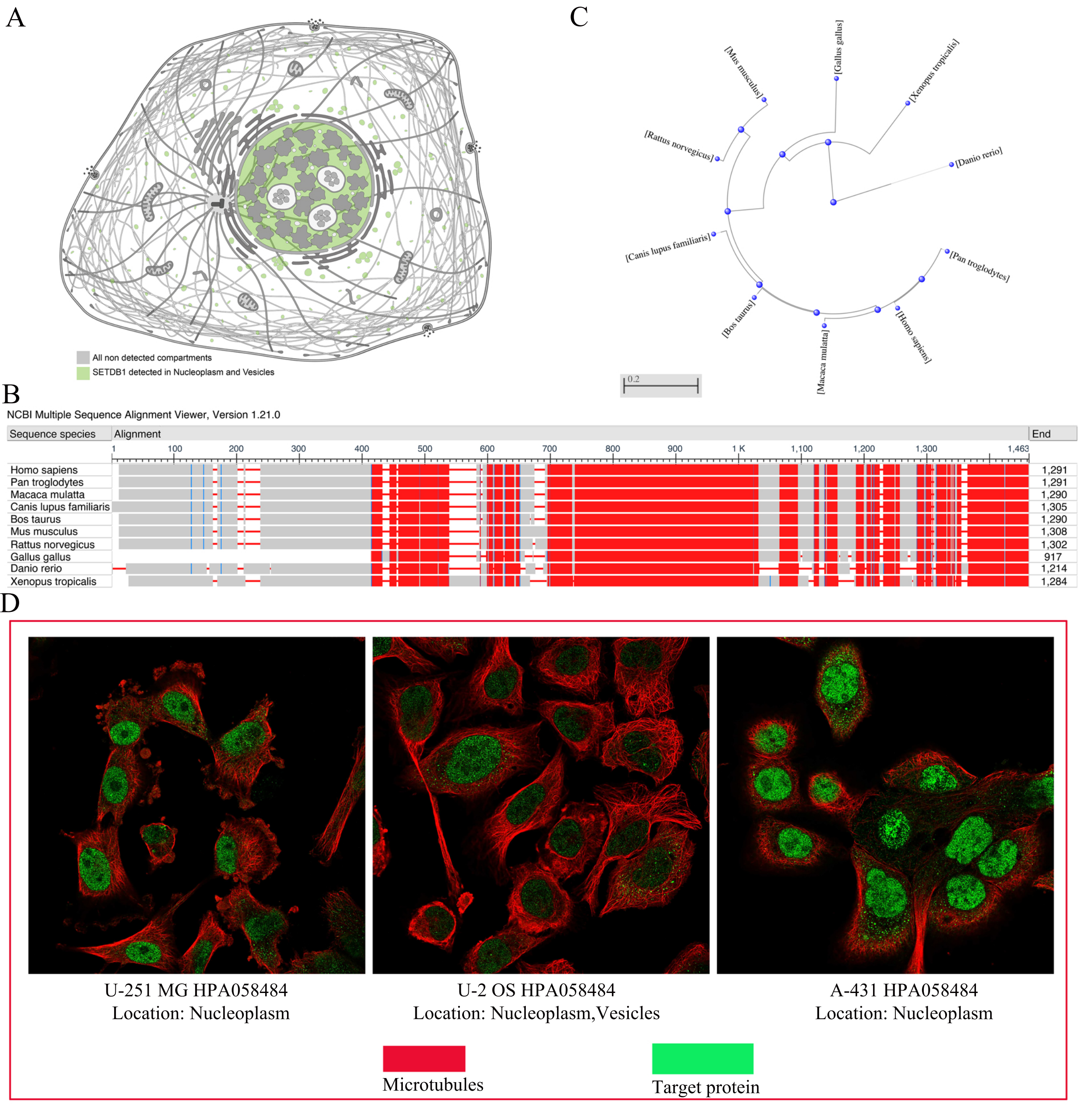
A
All non detected compartments
SETDB1 detected in Nucleoplasm and Vesicles
C
[Mus musculus]
[Gallus gallus]
[Xenopus tropicalis]
[Rattus norvegicus]
[Danio rerio]
[Canis lupus familiaris]
[Pan troglodytes]
[Bos taurus]
[Homo sapiens]
[Macaca mulatta]
0.2
B
NCBI Multiple Sequence Alignment Viewer, Version 1.21.0
Sequence species
Alignment
End
1
100
200
300
400
500
600
700
800
900
1 K
1,100
1,200
1,300
1,463
Homo sapiens 1,291
Pan troglodytes 1,291
Macaca mulatta 1,290
Canis lupus familiaris 1,305
Bos taurus 1,290
Mus musculus 1,308
Rattus norvegicus 1,302
Gallus gallus 917
Danio rerio 1,214
Xenopus tropicalis 1,284
D
U-251 MG HPA058484
Location: Nucleoplasm
U-2 OS HPA058484
Location: Nucleoplasm,Vesicles
A-431 HPA058484
Location: Nucleoplasm
Microtubules
Target protein
